# Language Network Connectivity of Euthymic Bipolar Patients Is Altered at Rest and during a Verbal Fluency Task

**DOI:** 10.3390/biomedicines11061647

**Published:** 2023-06-06

**Authors:** Zaira Romeo, Marco Marino, Dante Mantini, Alessandro Angrilli, Chiara Spironelli

**Affiliations:** 1 Department of General Psychology, University of Padova, 35131 Padova, Italy; 2 Movement Control and Neuroplasticity Research Group, KU Leuven, 3001 Leuven, Belgium; 3 Padova Neuroscience Center, University of Padova, 35131 Padova, Italy

**Keywords:** resting state, fluency task, fMRI, brain connectivity, language network, bipolar disorder

## Abstract

Abnormalities of the Language Network (LN) have been found in different psychiatric conditions (e.g., schizophrenia and bipolar disorder), supporting the hypothesis that language plays a central role in a high-level integration/connectivity of second-level cognitive processes and the underlying cortical regions. This view implies a continuum of shared neural alterations along the psychotic disorder spectrum. In particular, bipolar disorder (BD) patients were recently documented to have an altered LN asymmetry during resting state. The extent to which the LN architecture is altered and stable also during a language task has yet to be investigated. To address this question, we analyzed fMRI data recorded during an open-eyes resting state session and a silent verbal fluency task in 16 euthymic BD patients and 16 matched healthy controls (HC). Functional connectivity in the LN of both groups was computed using spatial independent component analysis, and group comparisons were carried out to assess the network organization during both rest and active linguistic task conditions. The LN of BD patients involved left and right brain areas during both resting state and linguistic task. Compared to the left-lateralized network found in HC, the BD group was characterized by two anterior clusters (in left frontal and right temporo-insular regions) and the disengagement of the posterior language areas, especially during the verbal fluency task. Our findings support the hypothesis that reduced language lateralization may represent a biological marker across different psychotic disorders and that the altered language network connectivity found at rest in bipolar patients is stable and pervasive as it is also impaired during a verbal fluency task.

## 1. Introduction

Over the last few years, the study of brain networks with neuroimaging techniques, such as functional magnetic resonance imaging (fMRI), has provided a deeper understanding of the neural substrates of cognitive functions [1]. This functional architecture of the brain has been explored in terms of both task-related and spontaneous activity [2,3]. Indeed, while several functional studies focused on brain responses elicited by specific stimuli or tasks, a large body of literature also reported coherent spatiotemporal patterns of neural activity, even in the absence of any external stimulation [2]. In particular, a growing number of studies focused on the identification of the relationship between this intrinsic network organization, as measured during resting state condition, and the one supporting active task. Notably, a series of contributions showed a relationship between resting state functional connectivity (FC) and task-evoked activations (e.g., [4,5,6]). For example, Tavor and colleagues used task-free acquisitions to predict task-evoked responses, including a large set of behavioral paradigms [4]. The results showed that task-free acquisitions were the best predictor of individual task-evoked activity across all tasks. Similarly, Cole et al. observed a significant statistical relationship between resting state and task-evoked FC, suggesting that large-scale intrinsic networks can shape specific activations during task [6].

Studies on the correspondence between task-dependent and task-free network topology in psychiatric conditions such as bipolar disorder (BD) are relatively scarce. Investigations in this direction might provide a link between a theoretical perspective (i.e., psychosis viewed as a continuum) and the future development of neurolinguistic markers of BD of clinical interest. BD is a psychiatric condition responsible for dysfunctional mood state, cognition, and emotional regulation [7,8]. It is characterized by a cyclical switch from depression to mania (BD-I) or hypomania (BD-II) [8,9]. During only the euthymic phase, BD patients do not exhibit significant clinical symptoms, although mood vulnerability is still present [10]. Network alterations in BD patients were widely documented during both task and rest conditions (for a review, see [11]). In particular, several experimental paradigms, such as executive function and working memory tasks, have been used to study network organization in BD patients and control adults [11,12,13,14,15,16,17,18,19,20]. Most of these studies reported BD alterations during both executive function and working memory tasks in the prefrontal areas and the dorsolateral regions in particular. Changes were also found in other regions, including the anterior cingulate cortex, frontopolar cortex, and parietal and ventral prefrontal cortices [18,19,20]. Notably, this large heterogeneity could be explained by differences in the experimental setup and task, the patients’ inclusion criteria, the implemented analysis, and the absence of control for confounding variables (e.g., the effects of medication). Concerning resting state studies, alterations were also found within different brain networks, including the default mode network (DMN), fronto-parietal network, salience network, central executive network, and language network (LN) [21,22,23,24,25,26,27,28], as well as in the functional connectivity between the cerebellum and temporal lobe regions of interests of lifetime hallucinating BD patients [29]. These differences either in network topology [28] or oscillatory activity [30] for the LN and the DMN, respectively, also emerged when considering BD patients in their euthymic phase. Interestingly, a relationship between network alterations and clinical scores, including maniac and depressive symptoms, was reported for both networks. This suggested that a psychobiological trace of BD patients’ vulnerability is still present in specific networks, such as DMN and LN, when frank symptomatology is also almost absent. Overall, the network approach provided useful markers for neurological and psychiatric disorders [31,32,33,34], offering great potential for diagnosis, treatment, and rehabilitation. Concerning BD, many studies investigated the presence of abnormalities in specific networks at rest or in response to a task and their correlations with clinical scales [18,19,28,30].

A recent study on euthymic BD patients showed alterations in the spatial organization of the LN, which, together with the typical left-lateralized area (i.e., the middle temporal gyrus), also included the right homologous regions (i.e., Broca and anterior insula) [28]. Indeed, for this network, BD patients recruited both left and right temporal areas. This finding is in line with Crow’s theory on the role of altered language lateralization at the origin of schizophrenia [35,36] and, more generally, functional psychoses [37]. Notably, Crow’s theory accounts for most of the psychotic symptoms, including delusions, auditory hallucinations, and thought disorders, all arising from the activation of two “uncertain” hemispheres. In this perspective, this model is easy to test, for example, by simply measuring hemispheric specialization for language. Beyond Crow’s original theory, considering that language lateralization is a *crystallized* characteristic of the human brain networking—acquired during childhood but persists for the rest of the individual’s life—a failure in this hemispheric specialization could represent a possible marker of mental disease, regardless of the current state of the patient. In other words, as language probably represents the most complex emerging property of a large and complex brain, any disorder that affects the delicate architecture and connectivity between and within hemispheres can, in principle, alter this equilibrium, thus explaining most of the symptoms and metalinguistic impairments which characterize the most severe psychiatric disorders (e.g., semantic anomalies, thought disorders, ruminations, and auditory hallucinations). Interestingly, structural and functional abnormalities in regions that are part of the language circuitry were reported, at the hemodynamic and electrophysiological level, both in schizophrenia patients with auditory verbal hallucinations [38,39,40,41], individuals with clinical high-risk psychosis [42], and bipolar [28] and major depressive (MDD) patients [43]. This evidence supports not only Crow’s original theory but also another recent model associated with functional psychoses, i.e., the continuum hypothesis of shared neural alterations in the psychotic spectrum disorders, including all these mental conditions characterized by an important difficulty in distinguishing what is real from what is not [44]. By studying the changes in the brain network organization (e.g., the recruitment of different brain regions), we could investigate the differences between intrinsic resting-related and task-evoked activity and their implications for the behavior. In particular, the focus on brain network reconfiguration from rest to task may reveal new insights into neural dysfunctions in the psychopathology of specific disorders. Indeed, as previously proposed, it is not clear whether FC abnormalities at rest could be associated with a similar abnormal response during active tasks [45,46]. Therefore, the execution of a task could allow probing the integrity of inter- and intra-hemispheric networks and processes within a specific cognitive domain.

In this present study, our goal was to investigate the influence of an engaging task on LN intrinsic organization. To this end, we aimed to analyze whether LN functional connectivity in euthymic BD patients is altered not only during resting state conditions but also when an active language task is performed. In particular, our hypothesis was that the euthymic condition is associated with functional abnormalities in a specific network (i.e., LN) not only during a resting state but also during a language-inducing task execution [45,46]. We tested this hypothesis on 16 BD patients in the euthymic phase and 16 healthy controls. In particular, we analyzed fMRI data recorded during an open-eyes resting state session and during a silent verbal fluency task, in which participants were asked to produce as many words as possible starting from a given letter. Our experimental design was oriented (i) to investigate the spontaneous organization of LN in both BD patients and healthy controls and (ii) to assess how the architecture of this network changes during a task involving language pathways. According to Crow’s theory [28,35,36,47] on psychosis origin and considering past results from a variety of clinical/subclinical samples (i.e., individuals with clinical high-risk for psychosis [42], BD [28], and MDD patients [43]), we expected that euthymic BD patients, despite their relatively stable condition, would recruit bilateral fronto-temporal LN areas during both rest and task conditions; in addition, we expected an enhanced effect of the task compared to the rest condition on the ability to ignite dysfunctional right homologous regions.

## 2. Materials and Methods

### 2.1. Participants

Sixteen euthymic BD patients (10 females, average age = 53.25 years, Standard Deviation [SD] = ±11.46 years) were recruited at the Mood Disorders Outpatient Unit of the Padova University Hospital and took part in this study. Patients were recruited according to the following inclusion criteria: (a) they received a diagnosis of bipolar disorder (type I or II) for at least one year; (b) they were non-remitting outpatients; (c) they were in a euthymic state at the moment of the experimental data collection (Young Mania Rating Scale [YMRS] scores being lower than 8) [48]. Details about anamnestic and clinical data of BD patients are summarized in Table 1. These patients represent a subgroup of the bipolar population investigated in previous works from our research group [28,30]. Sixteen healthy, age-matched, controls (HC) were also included in this study (average group means ± SD in Table 1).

We only recruited healthy participants, not reporting kinship with some members of the patient group, use of psychotropic drugs, and major lifetime psychiatric diagnosis. All participants, both BD patients and HCs, were suitable for MRI scanning and did not suffer from epilepsy or any other major neurologic brain comorbidities. This present study adheres to the principles of the Declaration of Helsinki and was approved by the Ethics Committee of Padua University Hospital. The informed written consent was signed by all participants before they started the experiment.

### 2.2. Clinical Assessment

All participants completed a psychiatric interview (Structured Clinical Interview for DSM-IV) before the MRI session with a board-certified psychiatrist to assess the presence or absence of current and past psychiatric illness. On the day of the experiment, a psychiatrist completed the YMRS as eligibility criterion, as well as the Hamilton Depression Rating Scale (HAM-D) [49], Altman Self-Rating Mania Scale (ASRM) [50], and STAI-Y1 and Y2 [51] with the BD participants. The higher the scores, the greater the amount of what was measured (e.g., depression, mania, or anxiety). Moreover, the Positive And Negative Affective State (PANAS) questionnaire [52] was also administered. Additional clinical information (i.e., pharmacological treatment, history of psychotic symptoms, age of onset of BD, duration, mood temporal pattern, and number of manic, hypomanic, or depressive episodes) is reported in Table 1.

### 2.3. MRI Data Acquisition

MRI data were acquired at the Radiology Department of Padua University Hospital with a Siemens MAGNETOM^®^ 1.5 T MRI system (Siemens Healthcare, Erlangen, Germany). MRI acquisition included (i) a resting state fMRI (rs-fMRI) scan during which participants were instructed to stay relaxed with their eyes open while focusing on a fixation cross which was presented in the center of the monitor, and not to think about anything in particular, while remaining motionless (151 continuous functional volumes, repetition time = 2390 ms, echo time = 50 ms, flip angle = 90°, field of matrix = 64 × 64 × 36, acquisition voxel size = 1.8 × 1.8 × 6 mm^3^; acquisition time 6:00 min) and (ii) a task-based fMRI acquisition, which was performed with the same acquisition parameters of the resting state scan. A high-resolution 3D T1-weighted structural MRI (sMRI) was also acquired using a gradient-echo sequence (160 sagittal slices, repetition time = 2000 ms, echo time = 3.13 ms, flip angle = 20°, field of matrix = 320 × 320 × 160, acquisition voxel size = 0.656 × 0.656 × 1 mm^3^; acquisition time 5:33 min). None of the subjects in this study reported anxiety or another particular discomfort during scanning or fell asleep. Following the MRI acquisition, all scans were visually inspected by a trained neuroradiologist to exclude gross pathology alterations, excessive motion, or major scanner artifacts. To quantify eventual head motion artifacts, we calculated the 151 framewise displacement [FD] [53] computed as the sum of the absolute values of the derivatives of the translational and rotational realignment estimates at every timepoint, for which we reported values below 0.5 for all groups and conditions (FD_HC_REST_ = 0.17 ± 0.1, FD_HC_TASK_ = 0.18 ± 0.11, FD_BD_REST_ = 0.26 ± 0.14, FD_BD_TASK_ = 0.28 ± 0.11).

### 2.4. Language Task

After the resting state acquisition, participants performed a silent verbal fluency task during scanning. The task consisted of a letter presented in the center of a monitor for two minutes, during which participants were asked to mentally produce as many noun words as possible starting with a given letter. This task was repeated three times, consecutively, with different letters, i.e., C, P, and S (total acquisition time 6:00 min). Participants were asked to be careful because the given letter would change from time to time. At the end of the silent task, we asked participants to orally repeat as many noun words as possible starting with the last letter “S” within the next 30 s. Immediately after the appearance of these written instructions, the experimenter warned, via microphone, that the participant could start.

The experimenter recorded each performance using a portable recording device. Offline, the recording was transcribed in the participant’s file. As a final step, two expert neuropsychologists independently rated the number of correctly generated words. There were no critical cases, as all the ratings corresponded.

### 2.5. MR Data Preprocessing

fMRI data were preprocessed via a standard automated procedure implemented in MATLAB (MathWorks, Natick, MA, USA) and based on SPM12 (http://www.fil.ion.ucl.ac.uk/spm/software/spm12, accessed on 1 September 2022), which we used in previous works from our research group [28,30]. This automated pipeline, which strictly follows the standard steps reported in SPM12 documentation (https://www.fil.ion.ucl.ac.uk/spm/doc/spm12_manual.pdf, accessed on 1 September 2022), included (i) motion correction for the functional image volumes, (ii) spatial alignment of the structural image to the functional data, and (iii) bias field correction for the functional data. These steps were all performed in native space. Following the (iv) co-registration of the functional data to MNI standard space using non-linear warping, (v) spatial smoothing of the functional data was finally performed with a 6 mm full-width half maximum [54,55]. For the normalization step, we used the standard Montreal Neurological Institute (MNI152) structural template image, adopted as the standard template by the International Consortium for Brain Mapping.

### 2.6. Functional Connectivity Analysis

For both the resting state and the task-based conditions, we performed functional connectivity analysis, separately for each subject, using spatial independent component analysis (ICA) [54,56]. This is a commonly used approach for resting state investigations, and here we also applied it to the silent fluency task, which we considered an “active” condition different from resting state. In particular, we used ICA to decompose the fMRI data into specific patterns, originating from independent sources, starting from the spatial covariance of the measured fMRI signals [56]. We estimated for each dataset the number of ICs via the minimum description length criterion [57]. This is a powerful method to achieve the best model using the shortest description of the data. For the resting state and the task-based scans, we extracted 35–75 ICs and 43–75 ICs, respectively, depending on the specific fMRI dataset. ICs were calculated using the Fast ICA algorithm proposed by Hyvarinen (1999) [58], with a deflation approach and hyperbolic tangent non-linearity [59]. This algorithm is implemented in MATLAB (MathWorks, Natick, MA, USA) and can be downloaded at the link http://research.ics.aalto.fi/ica/fastica/, accessed on 1 September 2022. For each IC, a spatial map and an associated time series were derived. In particular, these spatial maps express the intensity of the activity across the voxels of that pattern, whereas the time series correspond to their course over time [60,61]. For each IC, the spatial map was converted to z-scores by subtracting the average intensity across voxels and dividing the resulting map by the standard deviation across voxels. In this present study, our goal was to investigate the influence of an engaging task, such as the silent verbal fluency task, on LN intrinsic organization. We, therefore, focused on the ICs corresponding to the LN, which we expected to be the network mostly involved in the linguistic task described above. To select the independent component associated with the LN, in each subject and each scan, we used a resting state LN spatial template from a previous study [54]. This was performed twice for each subject, once for the ICs extracted from the resting state data and once for the ones extracted from the task-based data. The IC corresponding to the LN was identified using an automated template-matching procedure based on a linear regression between ICs and each template [54]. Each LN component, for each subject, was identified using a quantitative comparison with a template of the LN. The LN template was derived from the group-level map of the LN from a previous study [54]. The LN component was identified by selecting the IC with the highest spatial correlation with the LN template. Correlation values resulting from this template-matching procedure, and a few representative examples of individual LN spatial maps for each group and condition, are reported in the Supplementary Materials (Figure S1).

### 2.7. Statistical Analysis

For each subject, we extracted the IC corresponding to the LN from the data acquired during both rest and task, and we then performed group-level and comparative analyses to identify those regions that were recruited (or not) between the two conditions and between the two study groups. Following the estimation of the single-subject level LN spatial map, we derived the LN group-level correlation maps, for both the resting state and task-based conditions, by performing a one-sample t-test, using a mass-univariate analysis. With this approach, a significant correlation at the group level is associated, for each considered network, with a certain voxel, when this voxel displays a significant group effect. The significance level was corrected for multiple comparisons between single-subject z-score correlation maps using the Benjamini–Hochberg false discovery rate (BH-FDR) procedure. This procedure does not make any assumptions about sample dependency [62]. The significance threshold for the LN group-level correlation maps was set to *p* < 0.05, using BH-FDR correction. This was computed separately for each group, i.e., the HC and the BD groups, to visualize the average LN functional connectivity patterns for both the resting state and the task-based condition. We then performed, for each network, the comparison between the HC and the BD groups for both the resting state and the task-based conditions. This was performed via a two-sample *t*-test on the individual LN maps derived for the two groups to detect regional differences in the LN maps when the participants were resting and when they were performing the silent fluency task. For the task condition only, the number of generated words was included in the analysis as covariate. This was necessary because groups were different in the years of education, which was, in turn, correlated with the number of words recalled at the end of the experiment. In addition, a Generalized Linear Model (GLM) was used for the construction of a voxel-wise regressor including task performance as covariate (as well as task performance and education as covariates; see Figure S2 in the Supplementary Materials).

## 3. Results

### 3.1. Socio-Demographical and Clinical Data

No significant socio-demographical differences between HC and BD groups emerged (all t < 1.0), apart from education years which differed between groups (t_30_ = 2.53, *p* = 0.017). All BD patients were pharmacologically treated with mood stabilizers, atypical antipsychotics, antidepressants, and anxiolytics (Table 1; further details in [30]).

### 3.2. Fluency Task

No overall differences were found in terms of the number of generated words and stated at the end of the experiment (mean ± standard deviation: HC = 6.50 ± 2.85, BD = 5.87 ± 2.87; t_30_ = 0.62, ns). As healthy controls revealed more education years than BD patients (15.62 ± 3.96 vs. 12.06 ± 4.01 years, respectively), we aimed to test whether this difference affected the performance at the statistical level. As expected, we found a positive correlation between the years of education and the performance at the fluency task (i.e., number of generated words) (r_30_ = 0.42, *p* = 0.015). Accordingly, the higher the number of education years, the greater the number of words referred to after the silent verbal fluency task.

### 3.3. ICA LN Spatial Maps at Rest

**Figure 1:**
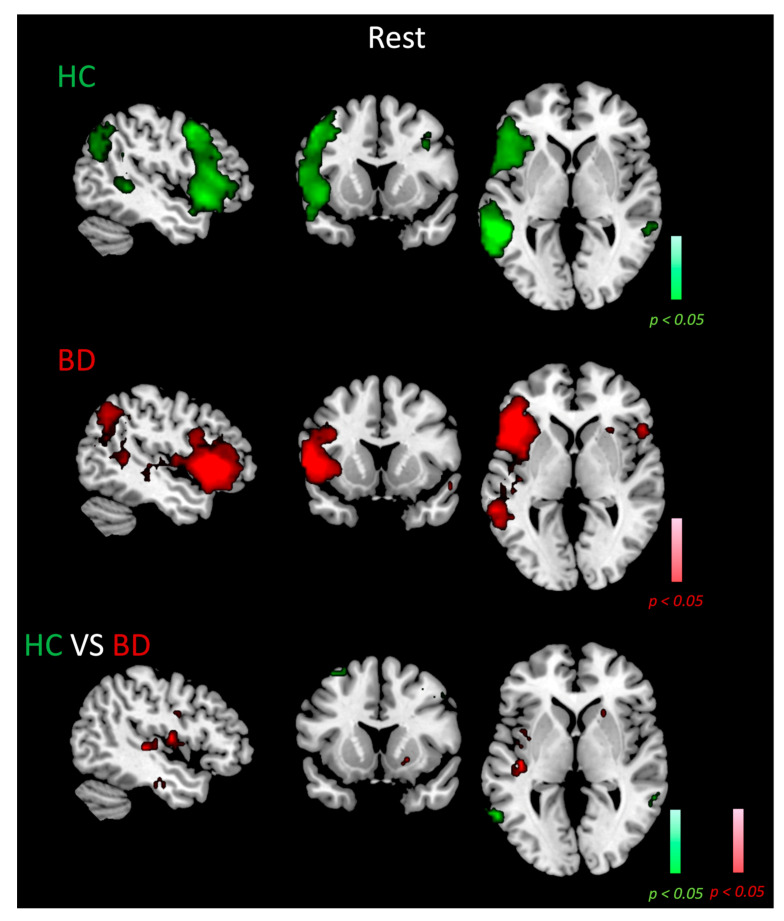
LN maps in HC and BD patients and contrast between groups during resting state. Random-effect group-level t-maps of the LN in HC (top row, green color scale) and BD patients (middle row, red color scale), and the random-effect group-level t-map for the difference between HC and BD patients (bottom row, green/red color scales depending on the group contrast), which was masked to only show the significant differences between the two groups for the LN areas and their homologous. All maps had a statistical threshold of *p* < 0.05, BH-FDR corrected.

Figure 1 depicts the random-effect group-level t-maps for LN of HC (top row) and BD patients (middle row) during resting state, as well as the random-effect group-level t-map for the difference between control and patient groups (bottom row). For both groups, the LN recruited typical areas in the left hemisphere, such as the Broca’s area/frontal operculum (BA44–45), insula (BA13), premotor and supplementary motor areas (BA6), angular gyrus (BA39), and superior and middle temporal gyrus (BA21–22) [60]. However, in the BD group, the LN also included the following homologous regions in the right hemisphere (Figure 1, second row, hot color scale): the insula (BA13), Broca’s area (BA45), the pars orbitalis (BA47), and the putamen. In the control group, we also observed the recruitment of regions in the right hemisphere, specifically in the middle temporal gyrus (BA21) and in the frontal eye fields (BA8).

The between-group analyses (third row of Figure 1, red and green color scale, for BD > HC and BD < HC contrasts) showed that BD had significantly higher connectivity compared to the control group in the right putamen (MNI coordinates: 22, 18, −1; the number of voxels: 14 voxels, t-score of the peak voxel in the significant cluster: −2.88) and right premotor/supplementary motor areas (BA6, MNI coordinates: 48, −4, 38, voxels: 19, t-score: −2.79). Greater connectivity for BD was also found in the left primary auditory area (BA41, MNI coordinates: −44, −24, 2, voxels: 22, t-score: −2.95), left insula (BA13, MNI coordinates: −41, 5, 2, voxels: 17, t-score: −2.84) and left premotor/supplementary motor areas (BA6, MNI coordinates: −45, −4, 8, voxels: 38, t-score: −3.7). Conversely, HC reported significantly higher connectivity in the right fusiform gyrus (BA37, MNI coordinates: 59, −47, 1, voxels: 34, t-score: 3.17) and left angular gyrus (BA39, MNI coordinates: −60, −42, 3, voxels: 33, t-score: 3.26).

### 3.4. ICA LN Spatial Maps during Fluency Task

**Figure 2:**
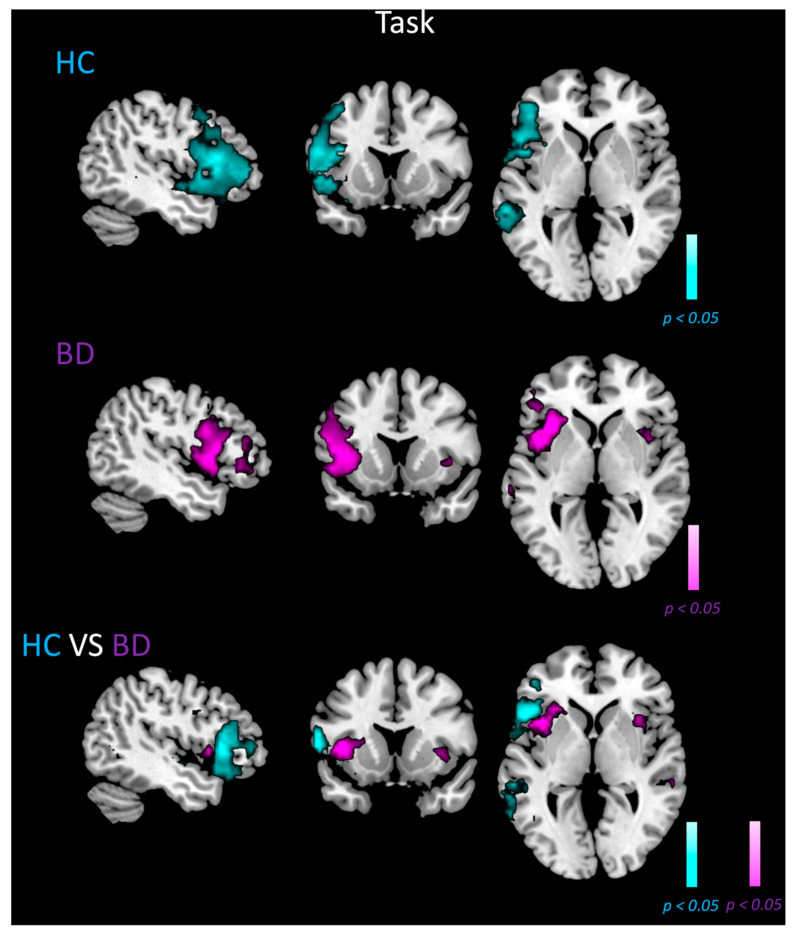
LN maps in HC and BD patients and contrast between groups during fluency task. Random-effect group-level t-maps of the LN in HC (top row, cyan color scale) and BD patients (middle row, purple color scale), and the random-effect group-level t-map for the difference between HC and BD patients (bottom row, cyan/purple color scales depending on the group contrast), which was masked to only show the significant differences between the two groups for the LN areas and their homologous. All maps had a statistical threshold of *p* < 0.05, BH-FDR corrected.

Figure 2 shows the random-effect group-level t-maps for LN of HC (top row) and BD patients (middle row) during the verbal fluency task, as well as the random-effects group-level t-map for the difference between the control and patient groups (bottom row). As for the resting state condition, during task execution, LN mainly recruited the typical left lateralized regions characteristic of this network (see, [60]). However, compared to the resting state, the verbal fluency task induced decreased connectivity in the left parietal areas in both HC and BD groups. This qualitative difference is supported by the random-effect group-level t-map for the difference between rest and task conditions for each group (see Figure S3 in the Supplementary Materials).

Compared with HC, BD patients showed lower recruitment of the left middle temporal gyrus, together with the concurrent recruitment of the right insula (BA13), right Broca’s area/ operculum (BA44), and right primary motor areas (BA4; Figure 2, middle row, purple color scale). No regions in the right hemisphere were activated for the control group (Figure 2, top row, cyan color scale).

The between-group analyses (third row of Figure 2, purple and cyan color scale, for BD > HC and BD < HC contrasts) showed that BD had significantly higher connectivity compared to healthy controls in the right insula (BA13, MNI coordinates: 35, 14, 2, voxels: 46, t-score: −4.14), right middle temporal gyrus (BA21, MNI coordinates: 61, −32, 0, voxels: 39, t-score: −3.78), and right superior temporal gyrus (BA22, MNI coordinates: 60, −30, 0, voxels: 45, t-score: −4.84), but also in the left insula (BA13, MNI coordinates: −37, 11, 0, voxels: 174, t-score: −5.63). The control group reported higher connectivity compared to BD patients in regions of the left hemisphere only, including Broca’s area (BA45, MNI coordinates: −49, 21, 0, voxels: 656, t-score: 5.91), middle temporal gyrus (BA21, MNI coordinates: −58, −34, 0, voxels: 246, t-score: 5.69), and pars orbitalis (BA47, MNI coordinates: −43, 24, 0, voxels: 53, t-score: 3.97).

## 4. Discussion

In this study, we investigated the influence of a verbal fluency task on LN organization in BD patients, aiming to investigate alterations of language lateralization [37] along the continuum of psychotic spectrum disorders [44,63]. A large body of literature examined the neural underpinnings of different psychopathologies, suggesting that abnormalities of language pathways lead to the emergence of psychoses [28,38,41,42,64]. In line with this account, schizophrenia patients exhibited (i) a decreased grey matter volume in regions that are part of the LN [38,41,65] and showed (ii) a failure of the typical left hemisphere dominance during linguistic tasks [40,47,66]. Functional alterations in the LN were also observed in BD patients in remission (i.e., in the euthymic phase), at least at rest [28]. Accordingly, we aimed to investigate whether alterations of LN functional connectivity characterize BD patients during the execution of a verbal fluency task. Indeed, an altered LN—bilaterally distributed—at rest could be functionally reactivated during the execution of a linguistic task that typically recruits a set of regions involved in language processing. To this end, we enrolled only patients in the euthymic phase to investigate whether the altered LN spatial distribution represents a stable trait neural correlate to bipolar disorder and, more generally, to a variety of psychotic disorders along the continuum.

The analysis of the LN at rest showed greater functional connectivity in BD compared to the healthy group in a right-lateralized cluster, including the putamen and the premotor/supplementary motor areas. The role of these regions in the processing of language information (e.g., production, naming, articulation) is well known. However, these functions are commonly associated with areas in the left, rather than in the right, hemisphere [67,68]. Our findings revealed that the LN of BD patients does not show the typical left-lateralized pattern but recruits more symmetrical clusters, including key homologous language regions in the right hemisphere. In line with the psychosis continuum hypothesis, this result is consistent with a recent meta-analysis on schizophrenia patients suffering from auditory verbal hallucinations that showed a decreased grey matter volume in left language regions (i.e., left insula and inferior frontal gyrus) in patients with respect to controls [41]. Indeed, structural atrophy in these brain areas might disrupt the typical left-hemispheric dominance for language [35,36] and contribute to the activation of two “uncertain” hemispheres rather than the dominant one. Consequently, the activity of the right hemisphere might be no more inhibited by the left one [28], thus explaining most of the symptoms and metalinguistic impairments characteristics of the most severe psychiatric disorders (e.g., semantic anomalies, thought disorders, ruminations, and auditory hallucinations).

BD patients also showed hyper-connectivity in the left hemisphere, particularly in the insula, primary auditory cortex, and premotor/supplementary motor areas. Therefore, the architecture of BD patients’ LN during the resting state apparently comprises a bilateral linguistic hub inhibiting more posterior regions. Indeed, we found a reduction in FC in the left temporal and parietal (i.e., left angular gyrus) and in right temporal regions (i.e., right fusiform gyrus) in the patients vs. control group. Notably, a series of studies revealed a relationship between the presence of auditory verbal hallucination in schizophrenia patients with altered connectivity within the temporo-parietal and the auditory cortices (for a review, see [64]). Furthermore, the hallucinatory phenomenon was also associated with increased symmetrical FC in frontal language regions [64]. As previously observed in schizophrenia cohorts, we found, also in bipolar patients in the euthymic phase, an altered LN configuration that could represent a trait marker explaining most of the symptoms and metalinguistic impairments typical of all severe psychiatric disorders distributed along the psychotic spectrum continuum.

Our aim was to assess the LN organization not only during the resting state but also during a task that typically elicits the activation of a left-lateralized circuitry [69]. On the basis of previous evidence highlighting the lack of language lateralization in BD at rest [28], we expected to observe a clear symmetry of the left and right sides during task execution. The results were in line with this assumption; compared to the HC group, BD patients showed greater connectivity in the right insula and right superior and middle temporal gyri. Importantly, as shown in Figure 2, the fluency task recruited a greater right cluster compared to the resting state condition. Moreover, the FC in the left insula was larger in BD compared to the control group. The insula is a key region that participates in several cognitive processes (e.g., language production, auditory, speech perception, sensory-motor integration, attention, and emotion) [70,71,72], and its dysfunctions were widely reported in schizophrenia patients with auditory verbal hallucinations [38,41,65,73]. The presence of similar neural alterations across different psychiatric illnesses supports the idea of the shared markers in the spectrum of psychotic disorders [63]. Consistent with our hypothesis, we observed a lack of asymmetry during the fluency task in BD patients, who recruited both key left and homologous right areas. Interestingly, an fMRI investigation has associated decreased language lateralization, specifically with psychosis [69]. In that study, psychotic patients with auditory verbal hallucinations, non-psychotic individuals with auditory verbal hallucinations, and healthy adults performed a silent verbal fluency task; no significant differences in brain activation were observed between non-psychotic voices hearers and healthy controls, while psychotic patients differed significantly from both these groups, showing increased activation of left insula and the right precentral gyrus.

Together with the loss of asymmetry, an important characteristic of our BD group is the additional alteration of the antero-posterior asymmetry of the LN. Within the left hemisphere, the control group reported greater frontal and temporal connectivity (i.e., Broca’s area, middle temporal gyrus, and pars orbitalis) compared to BD patients, whereas the latter did not show any involvement of the posterior temporal part. A possible explanation is that the increased FC of the two (left and right) frontal linguistic hubs in BD patients increases the inhibitory control of the posterior regions. Notably, the left middle temporal gyrus, showing higher FC in controls vs. BD, plays an important role in language processing [74]. Moreover, a recent study found decreased middle temporal gyrus connectivity in schizophrenia patients with auditory hallucinations [39]. In healthy brains, the language network comprises two main clusters: an anterior center specialized in speech production and a posterior one involved in language comprehension [75]. These clusters are highly interconnected within the left hemisphere, both at the cortical and subcortical levels, to ensure efficient language processing. We speculate that the hyper-connectivity of the anterior centers might inhibit the posterior portions of the temporal lobe in bipolar patients, also when there are no frank symptoms in the remitted patients (i.e., during euthymia). Past research using language tasks in euthymic BD patients is quite limited; nevertheless, Curtis et al. found abnormal prefrontal activation in patients compared to controls during semantic and phonetic tasks [76]. Specifically, they found no difference in the behavioral performance of BD patients compared with controls, together with greater left prefrontal activation (6, 44, and 45 BAs) during phonetic tasks and no differences in the right hemisphere in patients and healthy controls. Results are relatively in line with the greater activation we found during our fluency task in the left insula, also including a small posterior portion of left BA44. Our results are comparable with those of [76], but the following differences should be noted: (1) the phonetic tasks of [76] consisted of two tasks collapsed, i.e., verbal fluency (as in our study) and a rhyming task; (2) in the quoted study connectivity in the LN network was not investigated.

Another interesting finding is that in euthymic BD patients, we found similar results during resting state and task conditions. However, the anterior symmetrical distribution of the LN and the reduced posterior temporal connectivity were more enhanced during the verbal fluency task. In other words, the functional activation of linguistic circuits during the silent fluency task is not enough to restore the typical left hemisphere dominance in BD euthymic patients; rather, the altered LN spatial distribution represents a stable neural correlate of bipolar disorder and, arguably, of a variety of psychotic disorders along the continuum. The similar LN imbalance found at rest and during task execution is in line with the hypothesis that intrinsic brain activity can shape the network organization during a related task [6]. The importance of brain spontaneous activity is also supported by its link with clinical symptomatology. Several recent studies on neurological and psychiatric populations have found correlations between network disruption at rest and specific impairments/symptoms [26,30,34,46,77]. For example, resting-state LN abnormalities were found to be positively associated with residual mania and negatively correlated with depression in BD patients [28]. As previously proposed, the spontaneous brain organization is able to affect task-induced activations [6] and might represent an informative marker of healthy and pathological brain function.

This study has some limitations, such as the relatively small number of participants and the potential effect of pharmacological treatments. Most of our patients were taking medications (i.e., mood stabilizers, antipsychotics, antidepressants, and anxiolytics), which could alter the BOLD signal. However, we only included in our sample euthymic patients in a stable phase of the disorder. Future studies on larger samples should investigate the impact of long-term medication on brain networks involved in language processing. Additionally, by increasing statistical power thanks to adequate and balanced samples, as well as specific hypotheses on brain functioning, future exploratory whole-brain analyses might reveal further differences in functional connectivity between task and resting conditions and extend the applicability of this approach to other tasks and brain networks, also depending on patients’ clinical phenotype. In particular, the comparison between BD patients type I and type II appears critical to clarify the contribution of psychotic symptoms to alter the brain functional connectivity.

## 5. Conclusions

Our data provided new insights into the neural signatures of BD and showed how a fluency task alters the intrinsic organization of the LN. First, we found that the euthymic BD patients had reduced left dominance in the LN during both resting state and task conditions. Second, the configuration of the LN was different for the BD patients compared to the control group in both conditions, as BD patients recruited two symmetric frontal hubs, and disengaged left temporal regions, during task execution. Finally, LN alterations in BD supported the presence of a shared neural mechanism in the psychiatric continuum, which includes schizophrenia, bipolar disorder, and major depressive disorder [37,63,78]. Since language is at the top of all higher cognitive processes by integrating and connecting many associative brain regions, its disruption could explain the variety of manifestations of different psychotic disorders, namely the disorder of thoughts, distractibility, and impulsivity typical of BD. At the same time, it could also explain the elevated vulnerability of BD patients, apparently in remission, to relapse. In this perspective, LN dysconnectivity may be considered a promising trans-diagnostic marker for psychotic spectrum disorders and psychosis vulnerability in people at high risk. Additional studies are warranted to confirm the key role of LN in the psychoses and to correlate its FC to psychiatric symptoms as already performed in neurological patients [37,63,78].

## Figures and Tables

**Table 1:** Demographic, anamnestic, and clinical data.

	HC (*n* = 16)	BD (*n* = 16)	Statistics
**Age (years)**	51.19 ± 11.44	53.25 ± 11.46	*t*_30_ = −0.51
**Gender**	8 M/8 F	7 M/9 F	χ^2^_1_ = 0.12
**Height (m)**	1.69 ± 0.08	1.68 ± 0.09	*t*_30_ = 0.33
**Weight (kg)**	69.41 ± 11.21	72.25 ± 8.37	*t*_30_ = −0.81
		**Mean ± SD**	**Range** **(min–max)**
**BD type**		I (*n* = 9) and II (*n* = 7)	
**Psychotic symptoms** **Age at onset (years)**		*n* = 7 out of 9 BD-I 29.07 ± 13.89	18–65
**Episodes (*n*)**			
Manic		0.87 ± 0.92	0–2
Hypomanic		1.60 ± 1.64	0–6
Depressive		2.93 ± 2.22	1–9
**STAI-Y1**		37.63 ± 10.53	20–57
**STAI-Y2**		47.06 ± 8.69	31–59
**ASRM**		5.69 ± 4.51	0–13
**HAM-D**		5.56 ± 3.85	1–13
**MEDICATIONS**			
SSRI		*n* = 2	
SSNRI		*n* = 4	
SNRI		*n* = 1	
Atypical antipsychotic		*n* = 11	
Mood stabilizer/Benzodiazepine		*n* = 2/1	
Anticonvulsant drugs		*n* = 6	
Atypical tetracyclic antidepressant		*n* = 1	
Lithium carbonate		*n* = 3	

## Data Availability

The data that support the findings of this study are available on request from the corresponding author. The data are not publicly available due to privacy or ethical restrictions.

## Supplementary Materials

The following supporting information can be downloaded at: https://www.mdpi.com/article/10.3390/biomedicines11061647/s1, Figure S1: LN maps: Individual LN spatial maps of four representative subjects for both the HC (Subj1 and Subj7), and BD (Subj12, and Subj15) groups separately extracted in both rest and task conditions. Correlation values associated with the matching-procedure with the LN template are shown. The LN template is shown at the top of the figure. For the individual LN spatial maps, a threshold was manually set to allow a clearer visualization; Figure S2: LN maps: contrast between BD and HC groups during fluency task. Random-effects group-level t-map for the difference between HC and BD patients (cyan (HC)/purple (BD) color scales depending on the group contrast), which was masked to only show the significant differences between the two groups for the LN areas and their homologous. All maps had a statistical threshold of *p* < 0.05, BH-FDR corrected; Figure S3: LN maps: contrast between rest and task conditions for HC and BD groups. Random-effects group-level t-map for the difference between rest and task conditions for the HC (green (rest)/cyan (task)), and the BD groups (red (rest)/purple (task)). The t-maps were masked to only show the significant differences between the two conditions for the LN areas and their homologous. All maps had a statistical threshold of *p* < 0.05, BH-FDR corrected.

## References

[1] Park H.J., Friston K. (2013). Structural and functional brain networks: From connections to cognition. Science (80-).

[2] Fox M.D., Raichle M.E. (2007). Spontaneous fluctuations in brain activity observed with functional magnetic resonance imaging. Nat. Rev. Neurosci..

[3] Cole M.W., Bassett D.S., Power J.D., Braver T.S., Petersen S.E. (2014). Intrinsic and task-evoked network architectures of the human brain. Neuron.

[4] Tavor I., Parker Jones O., Mars R.B., Smith S.M., Behrens T.E., Jbabdi S. (2016). Task-free MRI predicts individual differences in brain activity during task performance. Science.

[5] Smith S.M., Fox P.T., Miller K.L., Glahn D.C., Fox P.M., Mackay C.E., Filippini N., Watkins K.E., Toro R., Laird A.R. (2009). Correspondence of the brain’s functional architecture during activation and rest. Proc. Natl. Acad. Sci. USA.

[6] Cole M.W., Ito T., Bassett D.S., Schultz D.H. (2016). Activity flow over resting-state networks shapes cognitive task activations. Nat. Neurosci..

[7] Qiu S., Chen F., Chen G., Jia Y., Gong J., Luo X., Zhong S., Zhao L., Lai S., Qi Z. (2019). Abnormal resting-state regional homogeneity in unmedicated bipolar II disorder. J. Affect. Disord..

[8] Lois G., Linke J., Wessa M. (2014). Altered functional connectivity between emotional and cognitive resting state networks in euthymic bipolar I disorder patients. PLoS ONE.

[9] Najt P., Hausmann M. (2014). Atypical right hemispheric functioning in the euthymic state of bipolar affective disorder. Psychiatry Res..

[10] Adler C.M., Holland S.K., Mills N.P., Delbello M.P., Eliassen J.C. (2005). Abnormal FMRI brain activation in euthymic bipolar disorder patients during a counting Stroop interference task. Am. J. Psychiatry.

[11] Vargas C., López-Jaramillo C., Vieta E. (2013). A systematic literature review of resting state network-functional MRI in bipolar disorder. J. Affect. Disord..

[12] Clark L., Sahakian B.J. (2008). Cognitive neuroscience and brain imaging in bipolar disorder. Dialogues Clin. Neurosci..

[13] Gruber O., Tost H., Henseler I., Schmael C., Scherk H., Ende G., Ruf M., Falkai P., Rietschel M. (2010). Pathological amygdala activation during working memory performance: Evidence for a pathophysiological trait marker in bipolar affective disorder. Hum. Brain Mapp..

[14] Kaladjian A., Jeanningros R., Azorin J.M., Nazarian B., Roth M., Mazzola-Pomietto P. (2009). Reduced brain activation in euthymic bipolar patients during response inhibition: An event-related fMRI study. Psychiatry Res. Neuroimaging.

[15] Keener M.T., Phillips M.L. (2007). Neuroimaging in bipolar disorder: A critical review of current findings. Curr. Psychiatry Rep..

[16] Lagopoulos J., Malhi G.S. (2007). A functional magnetic resonance imaging study of emotional Stroop in euthymic bipolar disorder. Neuroreport.

[17] Lagopoulos J., Ivanovski B., Malhi G.S. (2007). An event-related functional MRI study of working memory in euthymic bipolar disorder. J. Psychiatry Neurosci..

[18] Monks P.J., Thompson J.M., Bullmore E.T., Suckling J., Brammer M.J., Williams S.C.R., Simmons A., Giles N., Lloyd A.J., Harrison C.L. (2004). A functional MRI study of working memory task in euthymic bipolar disorder: Evidence for task-specific dysfunction. Bipolar Disord..

[19] Wessa M., Houenou J., Paillère-Martinot M.-L., Berthoz S., Artiges E., Leboyer M., Martinot J.-L. (2007). Fronto-striatal overactivation in euthymic bipolar patients during an emotional go/nogo task. Am. J. Psychiatry.

[20] Liu C.H., Li F., Li S.F., Wang Y.J., Tie C.L., Wu H.Y., Zhou Z., Zhang D., Dong J., Yang Z. (2012). Abnormal baseline brain activity in bipolar depression: A resting state functional magnetic resonance imaging study. Psychiatry Res. Neuroimaging.

[21] Liu C.H., Ma X., Wu X., Zhang Y., Zhou F.C., Li F., Tie C.L., Dong J., Wang Y.J., Yang Z. (2013). Regional homogeneity of resting-state brain abnormalities in bipolar and unipolar depression. Prog. Neuro-Psychopharmacol. Biol. Psychiatry.

[22] Liu Y., Wu X., Zhang J., Guo X., Long Z., Yao L. (2015). Altered effective connectivity model in the default mode network between bipolar and unipolar depression based on resting-state fMRI. J. Affect. Disord..

[23] Zeng C., Ross B., Xue Z., Huang X., Wu G., Liu Z., Tao H., Pu W. (2021). Abnormal Large-Scale Network Activation Present in Bipolar Mania and Bipolar Depression Under Resting State. Front. Psychiatry.

[24] Bellani M., Bontempi P., Zovetti N., Gloria Rossetti M., Perlini C., Dusi N., Squarcina L., Marinelli V., Zoccatelli G., Alessandrini F. (2020). Resting state networks activity in euthymic bipolar disorder. Bipolar Disord..

[25] Mamah D., Barch D.M., Repovš G. (2013). Resting state functional connectivity of five neural networks in bipolar disorder and schizophrenia. J. Affect. Disord..

[26] Jeganathan J., Perry A., Bassett D.S., Roberts G., Mitchell P.B., Breakspear M. (2018). Fronto-limbic dysconnectivity leads to impaired brain network controllability in young people with bipolar disorder and those at high genetic risk. NeuroImage Clin..

[27] Doucet G.E., Bassett D.S., Yao N., Glahn D.C., Frangou S. (2017). The role of intrinsic brain functional connectivity in vulnerability and resilience to bipolar disorder. Am. J. Psychiatry.

[28] Romeo Z., Marino M., Angrilli A., Semenzato I., Favaro A., Magnolfi G., Padovan G., Mantini D., Spironelli C. (2022). Altered language network lateralization in euthymic bipolar patients: A pilot study. Transl. Psychiatry.

[29] Hwang M., Roh Y.S., Talero J., Cohen B.M., Baker J.T., Brady R.O., Öngür D., Shinn A.K. (2021). Auditory hallucinations across the psychosis spectrum: Evidence of dysconnectivity involving cerebellar and temporal lobe regions. NeuroImage Clin..

[30] Marino M., Romeo Z., Angrilli A., Semenzato I., Favaro A., Magnolfi G., Padovan G., Mantini D., Spironelli C. (2021). Default mode network shows alterations for low-frequency fMRI fluctuations in euthymic bipolar disorder. J. Psychiatr. Res..

[31] Fornito A., Zalesky A., Breakspear M. (2015). The connectomics of brain disorders. Nat. Rev. Neurosci..

[32] Lynall M.E., Bassett D.S., Kerwin R., McKenna P.J., Kitzbichler M., Muller U., Bullmore E. (2010). Functional connectivity and brain networks in schizophrenia. J. Neurosci..

[33] Siegel J.S., Ramsey L.E., Snyder A.Z., Metcalf N.V., Chacko R.V., Weinberger K., Baldassarre A., Hacker C.D., Shulman G.L., Corbetta M. (2016). Disruptions of network connectivity predict impairment in multiple behavioral domains after stroke. Proc. Natl. Acad. Sci. USA.

[34] Romeo Z., Mantini D., Durgoni E., Passarini L., Meneghello F., Zorzi M. (2021). Electrophysiological signatures of resting state networks predict cognitive deficits in stroke. Cortex.

[35] Crow T.J. (1997). Schizophrenia as failure of hemispheric dominance for language. Trends Neurosci..

[36] Crow T.J. (2000). Schizophrenia as the price that Homo sapiens pays for language: A resolution of the central paradox in the origin of the species. Brain Res. Interact..

[37] Crow T.J. (2008). The “big bang” theory of the origin of psychosis and the faculty of language. Schizophr. Res..

[38] Modinos G., Costafreda S.G., van Tol M., McGuire P.K., Aleman A., Allen P. (2013). Neuroanatomy of auditory verbal hallucinations in schizophrenia: A quantitative meta-analysis of voxel-based morphometry studies Gemma. Cortex.

[39] Zhang L., Li B., Wang H., Li L., Liao Q., Liu Y., Bao X., Liu W., Yin H., Lu H. (2017). Decreased middle temporal gyrus connectivity in the language network in schizophrenia patients with auditory verbal hallucinations. Neurosci. Lett..

[40] Spironelli C., Angrilli A., Stegagno L. (2008). Failure of language lateralization in schizophrenia patients: An ERP study on early linguistic components. J. Psychiatry Neurosci..

[41] Romeo Z., Spironelli C. (2022). Hearing voices in the head: Two meta -analyses on structural correlates of auditory hallucinations in schizophrenia. NeuroImage Clin..

[42] Sabb F.W., van Erp T.G.M., Hardt M.E., Dapretto M., Caplan R., Cannon T.D., Bearden C.E. (2010). Language network dysfunction as a predictor of outcome in youth at clinical high risk for psychosis. Schizophr. Res..

[43] Spironelli C., Maffei A., Romeo Z., Piazzon G., Padovan G., Magnolfi G., Pasini I., Gomez Homen F., Concari G., Angrilli A. (2020). Evidence of language-related left hypofrontality in Major Depression: An EEG Beta band study. Sci. Rep..

[44] Craddock N., O’Donovan M.C., Owen M.J. (2009). Psychosis genetics: Modeling the relationship between schizophrenia, bipolar disorder, and mixed (or “schizoaffective”) psychoses. Schizophr. Bull..

[45] He B.J., Snyder A.Z., Vincent J.L., Epstein A., Shulman G.L., Corbetta M. (2007). Breakdown of Functional Connectivity in Frontoparietal Networks Underlies Behavioral Deficits in Spatial Neglect. Neuron.

[46] Baldassarre A., Ramsey L.E., Hacker C.L., Callejas A., Astafiev S.V., Metcalf N.V., Zinn K., Rengachary J., Snyder A.Z., Carter A.R. (2014). Large-scale changes in network interactions as a physiological signature of spatial neglect. Brain.

[47] Angrilli A., Spironelli C., Elbert T., Crow T.J., Marano G., Stegagno L. (2009). Schizophrenia as failure of left hemispheric dominance for the phonological component of language. PLoS ONE.

[48] Young R.C., Biggs J.T., Ziegler V.E., Meyer D.A. (1978). A rating scale for mania: Reliability, validity and sensitivity. Br. J. Psychiatry.

[49] Hamilton M. (1960). A rating scale for depression. J. Neurol. Neurosurg. Psychiat..

[50] Altman E.G., Hedeker D., Peterson J.L., Davis J.M. (1997). The altman self-rating Mania scale. Biol. Psychiatry.

[51] Pedrabissi L., Santinello M. (1989). State-Trait Anxiety Inventory—Form Y. Ital. Vers..

[52] Watson D., Clark L.A., Tellegen A. (1988). Development and Validation of Brief Measures of Positive and Negative Affect: The PANAS Scales. J. Pers. Soc. Psychol..

[53] Power J.D., Barnes K.A., Snyder A.Z., Schlaggar B.L., Petersen S.E. (2012). Spurious but systematic correlations in functional connectivity MRI networks arise from subject motion. Neuroimage.

[54] Mantini D., Corbetta M., Romani G.L., Orban G.A., Vanduffel W. (2013). Evolutionarily Novel Functional Networks in the Human Brain?. J. Neurosci..

[55] Marino M., Arcara G., Porcaro C., Mantini D. (2019). Hemodynamic Correlates of Electrophysiological Activity in the Default Mode Network. Front. Neurosci..

[56] McKeown M.J., Makeig S., Brown G.G., Jung T.P., Kindermann S.S., Bell A.J., Sejnowski T.J. (1998). Analysis of fMRI data by blind separation into independent spatial components. Hum. Brain Mapp..

[57] Calhoun V.D., Adali T., Pearlson G.D., Pekar J.J. (2001). Spatial and temporal independent component analysis of functional MRI data containing a pair of task-related waveforms. Hum. Brain Mapp..

[58] Hyvärinen A. (1999). Fast and robust fixed-point algorithms for independent component analysis. IEEE Trans. Neural Netw..

[59] Esposito F., Scarabino T., Hyvarinen A., Himberg J., Formisano E., Comani S., Tedeschi G., Goebel R., Seifritz E., Di Salle F. (2005). Independent component analysis of fMRI group studies by self-organizing clustering. Neuroimage.

[60] Mantini D., Perrucci M.G., Del Gratta C., Romani G.L., Corbetta M. (2007). Electrophysiological signatures of resting state networks in the human brain. Proc. Natl. Acad. Sci. USA.

[61] Mantini D., Franciotti R., Romani G.L., Pizzella V. (2008). Improving MEG source localizations: An automated method for complete artifact removal based on independent component analysis. Neuroimage.

[62] Benjamini Y., Hochberg Y. (1995). Controlling the False Discovery Rate: A Practical and Powerful Approach to Multiple Testing. J. R. Stat. Soc. Ser. B.

[63] Sorella S., Lapomarda G., Messina I., Frederickson J.J., Siugzdaite R., Job R., Grecucci A. (2019). Testing the expanded continuum hypothesis of schizophrenia and bipolar disorder. Neural and psychological evidence for shared and distinct mechanisms. NeuroImage Clin..

[64] Ćurčić-Blake B., Ford J.M., Hubl D., Orlov N.D., Sommer I.E., Waters F., Allen P., Jardri R., Woodruff P.W., David O. (2017). Interaction of language, auditory and memory brain networks in auditory verbal hallucinations. Prog. Neurobiol..

[65] Palaniyappan L., Balain V., Radua J., Liddle P.F. (2012). Structural correlates of auditory hallucinations in schizophrenia: A meta-analysis. Schizophr. Res..

[66] Spironelli C., Angrilli A. (2015). Language-related gamma EEG frontal reduction is associated with positive symptoms in schizophrenia patients. Schizophr. Res..

[67] Viñas-Guasch N., Wu Y.J. (2017). The role of the putamen in language: A meta-analytic connectivity modeling study. Brain Struct. Funct..

[68] Sun H., Zheng D., Wang X., Lu Z., Theysohn N., Forsting M., Guo Q. (2013). Functional segregation in the left premotor cortex in language processing: Evidence from fMRI. J. Integr. Neurosci..

[69] Diederen K.M., De Weijer A.D., Daalman K., Blom J.D., Neggers S.F.W., Kahn R.S., Sommer I.E.C. (2010). Decreased language lateralization is characteristic of psychosis, not auditory hallucinations. Brain.

[70] Bamiou D.E., Musiek F.E., Luxon L.M. (2003). The insula (Island of Reil) and its role in auditory processing: Literature review. Brain Res. Rev..

[71] Oh A., Duerden E.G., Pang E.W. (2014). The role of the insula in speech and language processing. Brain Lang..

[72] Uddin L.Q., Nomi J.S., Hebert-Seropian B., Ghaziri J., Boucher O. (2017). Structure and function of the human insula Lucina. J. Clin. Neurophysiol..

[73] Wylie K.P., Tregellas J.R. (2010). The role of the insula in schizophrenia. Schizophr. Res..

[74] Whitney C., Jefferies E., Kircher T. (2011). Heterogeneity of the left temporal lobe in semantic representation and control: Priming multiple versus single meanings of ambiguous words. Cereb. Cortex.

[75] Spironelli C., Angrilli A. (2015). Brain plasticity in aphasic patients: Intra- and inter-hemispheric reorganisation of the whole linguistic network probed by N150 and N350 components. Sci. Rep..

[76] Curtis V.A., Thompson J.M., Seal M.L., Monks P.J., Lloyd A.J., Harrison L., Brammer M.J., Williams S.C.R., Murray R.M., Young A.H. (2007). The nature of abnormal language processing in euthymic bipolar I disorder: Evidence for a relationship between task demand and prefrontal function. Bipolar Disord..

[77] Siegel J.S., Shulman G.L., Corbetta M. (2022). Mapping correlated neurological deficits after stroke to distributed brain networks. Brain Struct. Funct..

[78] Spironelli C., Romeo Z., Maffei A., Angrilli A. (2019). Comparison of automatic visual attention in schizophrenia, bipolar disorder, and major depression: Evidence from P1 event-related component. Psychiatry Clin. Neurosci..

