# Supplementary material for: Language Network Connectivity of Euthymic Bipolar Patients Is Altered at Rest and during a Verbal Fluency Task
Source: Biomedicines. 2023 Jun 6;11(6):1647. doi: 10.3390/biomedicines11061647 (PMC10296300; doi:10.3390/biomedicines11061647)
Supplement: Supplementary file 1 [file biomedicines-11-01647-s001.zip › biomedicines-2288952-supplementary.pdf]

Supplementary Materials for

Language network connectivity of euthymic bipolar patients is altered at rest and during verbal fluency task

Zaira Romeo[1], Marco Marino[1,2], Dante Mantini[2], Alessandro Angrilli[1,3] and Chiara Spironelli[1,3,*]

[1] Department of General Psychology, University of Padova, Italy
[2] Movement Control and Neuroplasticity Research Group, KU, Leuven, Belgium
[3] Padova Neuroscience Center, University of Padova, Italy
*Correspondence: chiara.spironelli@unipd.it; Tel.: +39 049 8276619

Individual ICA LN spatial maps

The independent component (IC) corresponding to the language network (LN) was separately extracted for each subject and for each condition following the application of the ICA algorithm. The IC corresponding to the LN was identified following a template-matching procedure by selecting the IC with the highest spatial correlation with a LN template, derived from the group-level map of the LN from a previous study (Mantini et al., 2013). The correlation values derived from this template-matching procedure were, for each group and for each condition, $rho_{HC\_REST} = 0.23 \pm 0.09$, $rho_{HC\_TASK} = 0.22 \pm 0.07$, $rho_{BD\_REST} = 0.27 \pm 0.08$, and $rho_{BD\_TASK} = 0.26 \pm 0.1$. A few examples of the individual LN spatial maps separately extracted for each subject and for each condition, and the template LN maps used for the matching procedure, are shown in Figure S1.

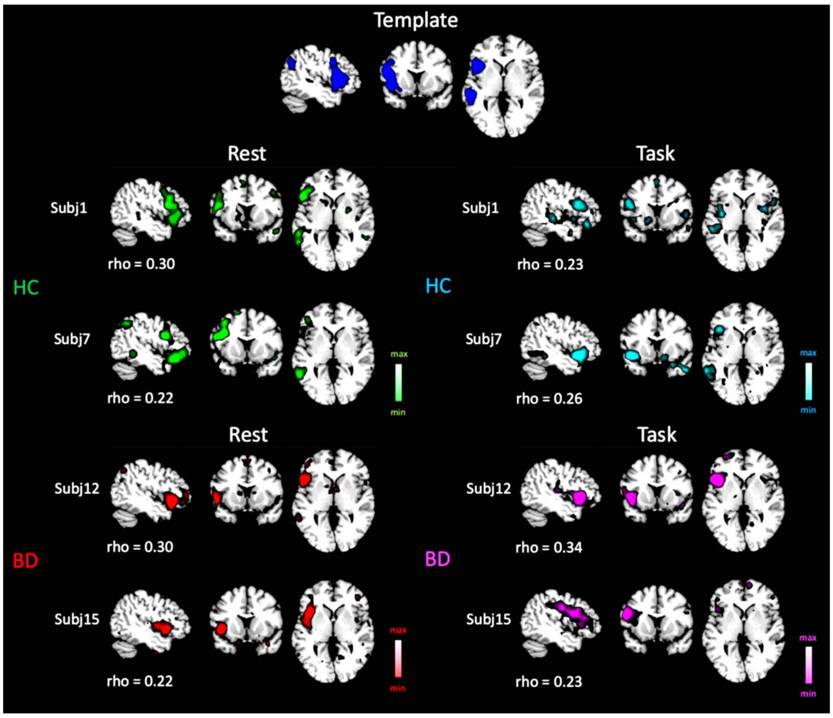

Figure S1. LN maps: Individual LN spatial maps of four representative subjects for both the HC (Subj1 and Subj7), and BD (Subj12, and Subj15) groups separately extracted in both rest and task conditions. Correlation values associated with the matching-procedure with the LN template are shown. The LN template is shown at the top of the figure. For the individual LN spatial maps, a threshold was manually set to allow a clearer visualization.

ICA LN spatial maps during fluency task – supplementary analysis I

For the task condition only, the task performance (i.e., the number of generated words soon after the silent fluency task) was included in the main analysis as covariate (Figure 2 in the main text of the manuscript). As education level and the silent task *performance* were positively correlated (r_{30} = .42, p = 0.015, ibidem) and groups differed for years of education, we decided to carry out a supplementary analysis to compare the LN spatial distribution between bipolar disorder (BD) patients and healthy controls (HC) controlling for the effect of two covariates: the number of words generated soon after the silent fluency task, and the education level. The results confirmed the pattern of results obtained when controlling for the number of words only (see Figure 2 in the manuscript and Figure S2). Relative to controls, BD patients activated two bilateral clusters. On the contrary, HC only activated left-lateralized regions.

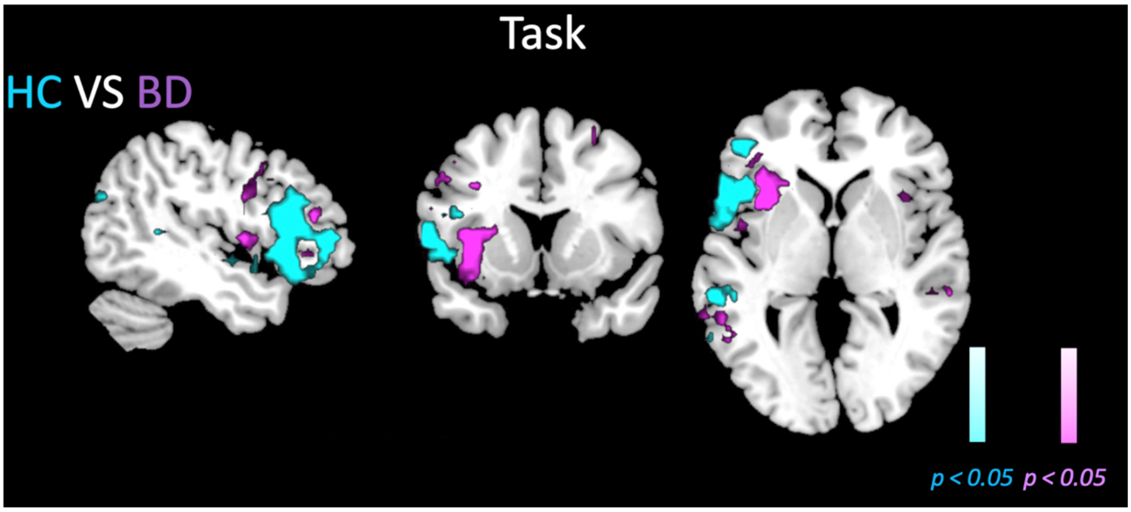

Figure S2. LN maps: contrast between BD and HC groups during fluency task. Random-effects group-level t-map for the difference between HC and BD patients (cyan (HC)/purple (BD) color scales depending on the group contrast), which was masked to only show the significant differences between the two groups for the LN areas and their homologous. All maps had a statistical threshold of $p < 0.05$, BH-FDR corrected.

ICA LN spatial maps: rest vs. fluency task – supplementary analysis II

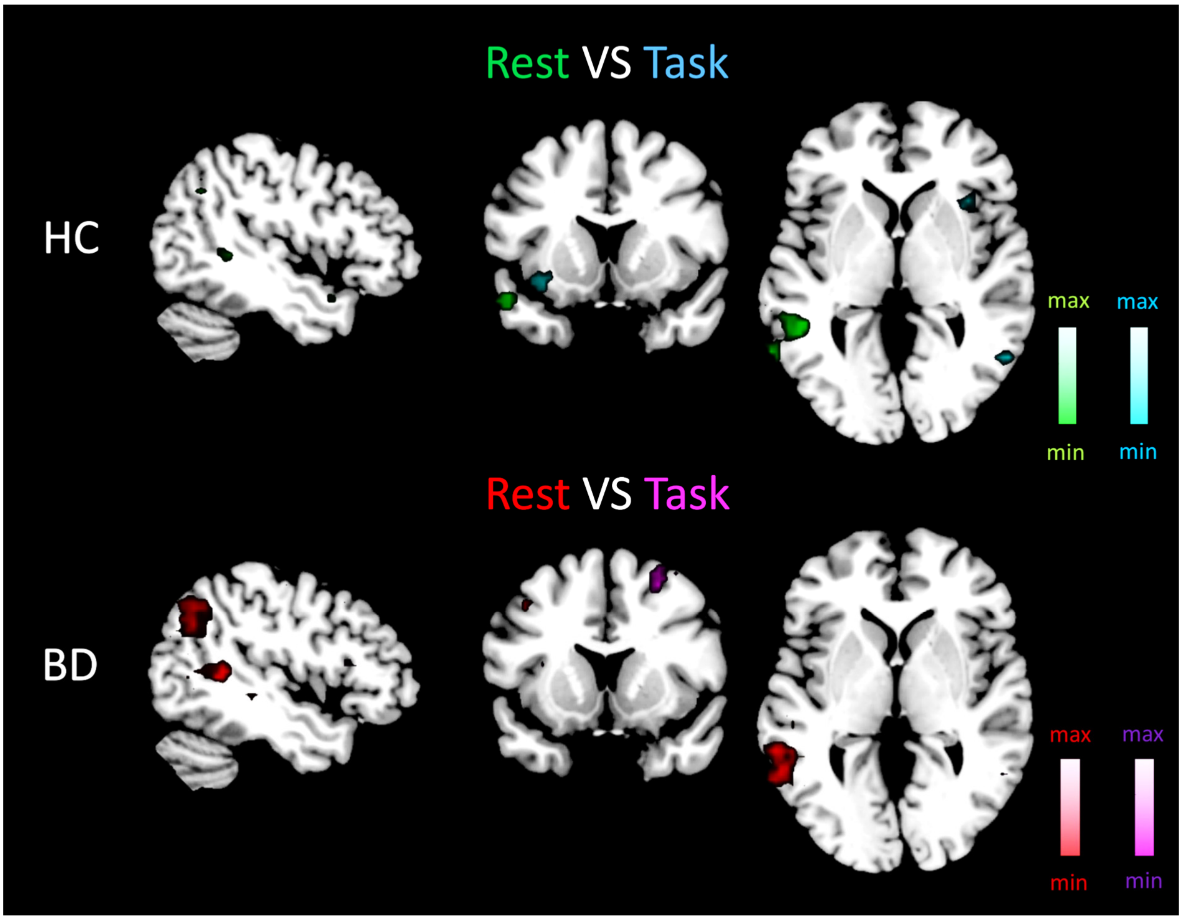

Figure S3. LN maps: contrast between rest and task conditions for HC and BD groups. Random-effects group-level t-map for the difference between rest and task conditions for the HC (green (rest)/cyan (task)), and the BD groups (red (rest)/purple (task)). The t-maps were masked to only show the significant differences between the two conditions for the LN areas and their homologous. All maps had a statistical threshold of $p < 0.05$, BH-FDR corrected.

REFERENCES

Mantini, D.; Corbetta, M.; Romani, G. L.; Orban, G. A.; Vanduffel, W. Evolutionarily Novel Functional Networks in the Human Brain? J. Neurosci. 2013, 33, 3259–3275, doi:10.1523/JNEUROSCI.4392-12.2013.
